# Supplementary material for: The clinicopathologic significance of Tks5 expression of peritoneal mesothelial cells in gastric cancer patients
Source: PLoS One. 2021 Jul 13;16(7):e0253702. doi: 10.1371/journal.pone.0253702 (PMC8277061; doi:10.1371/journal.pone.0253702)
Supplement: S1 Fig — A, Representative images of peritoneal mesothelial cells in healthy cases. The weak expression of Tks5 was found in the cytoplasm of the monolayer PMCs of the healthy peritoneum. Arrow, PMC: peritoneal mesothelial cell. B, Representative images of peritoneal mesothelial cells in gastric cancer cases. The Calretinin expression was found at PMCs with Tks5 expression the surface of peritoneum of gastric cancer cases. Arrow, PMC: peritoneal mesothelial cell. (PPTX) [file pone.0253702.s001.pptx]

## Slide 1
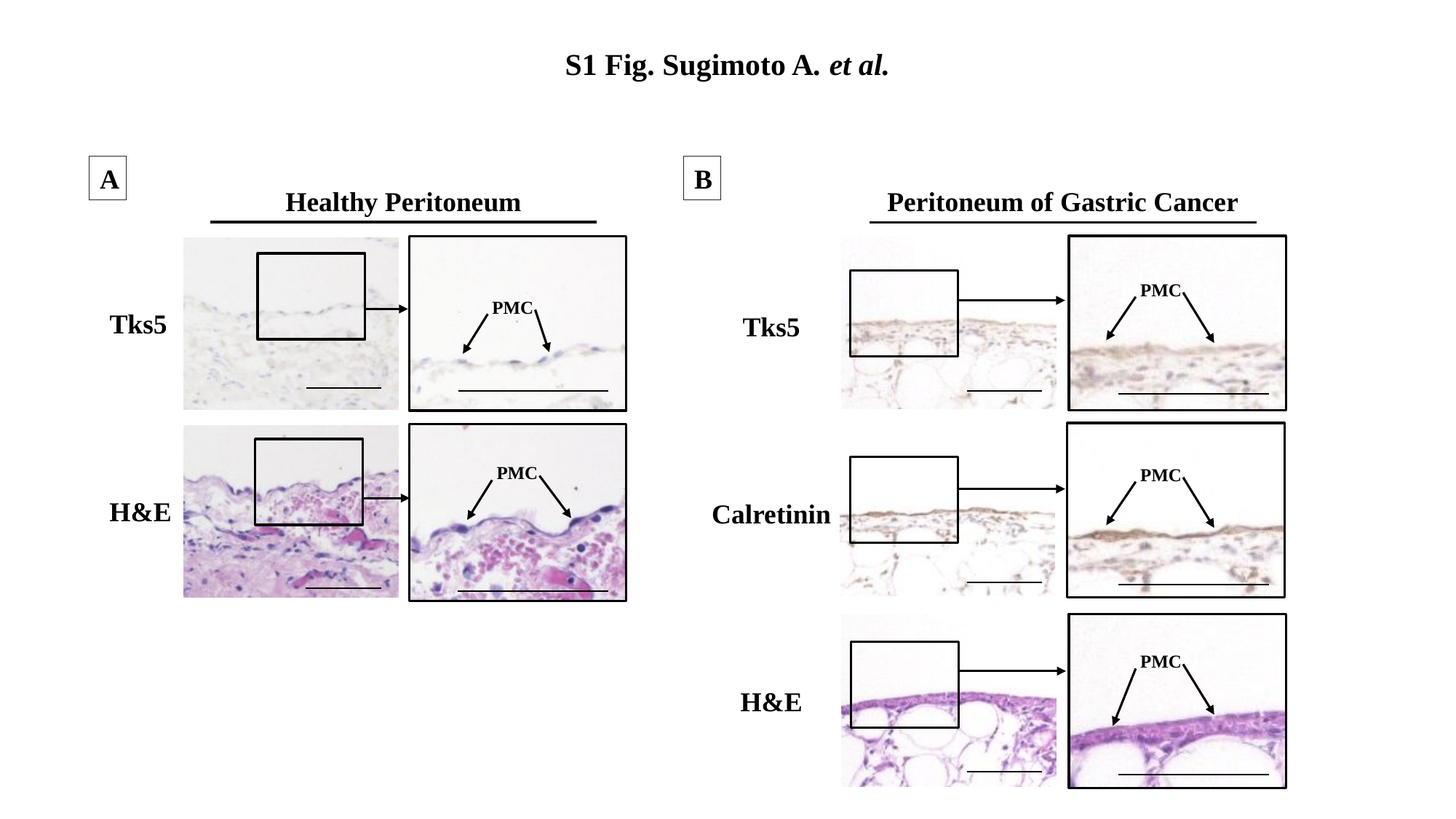

S1 Fig. Sugimoto A. et al.
A
B
Healthy Peritoneum
Peritoneum of Gastric Cancer
PMC
PMC
Tks5
Tks5
PMC
PMC
H&E
Calretinin
PMC
H&E
