# Supplementary material for: The clinicopathologic significance of Tks5 expression of peritoneal mesothelial cells in gastric cancer patients
Source: PLoS One. 2021 Jul 13;16(7):e0253702. doi: 10.1371/journal.pone.0253702 (PMC8277061; doi:10.1371/journal.pone.0253702)
Supplement: S2 Fig — No significant difference was found between the Tks5-positive and -negative groups in all patients (A; n = 110) and patients with R0 curative operation (B; n = 98). (PPTX) [file pone.0253702.s002.pptx]

## Slide 1
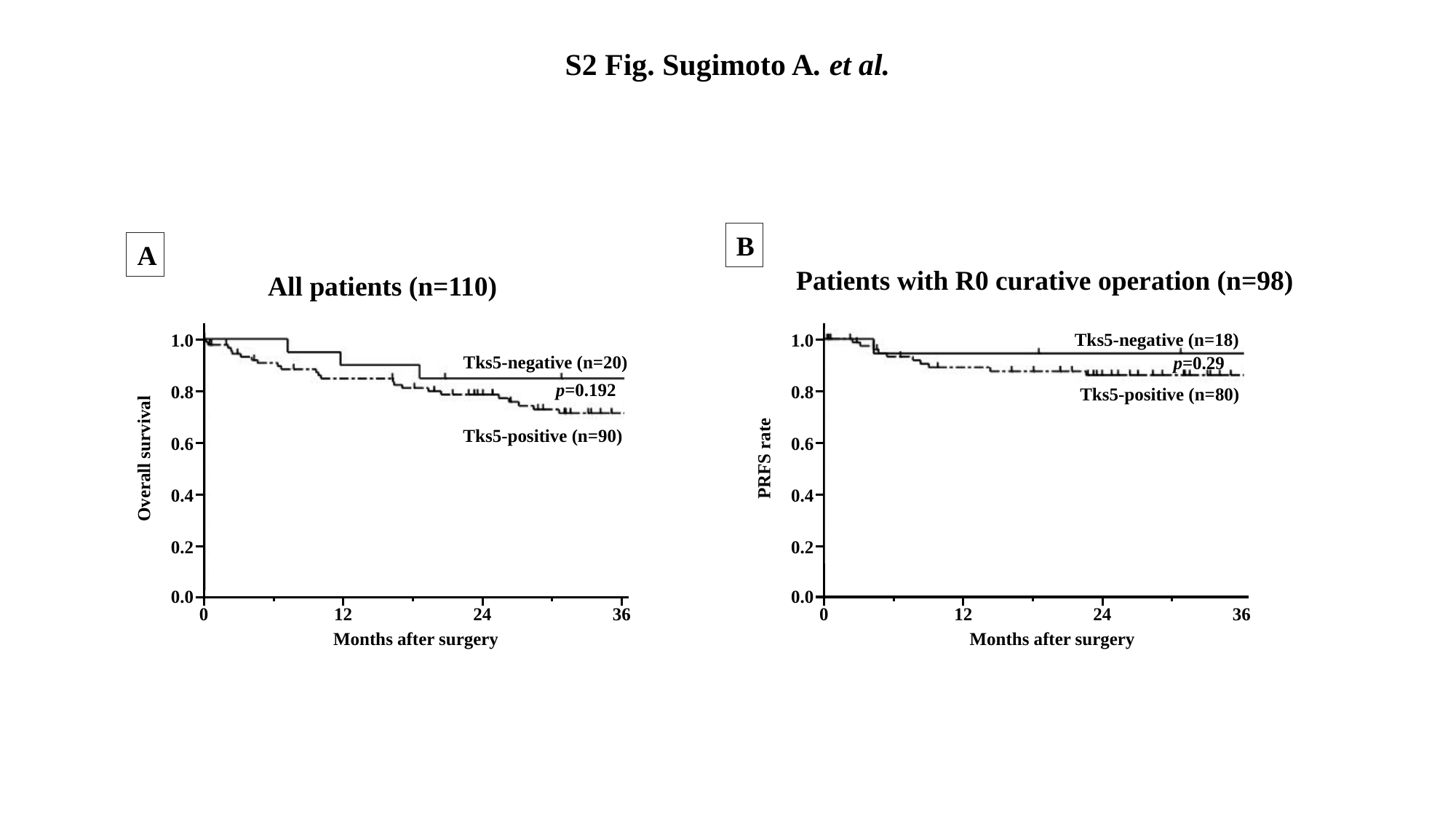

S2 Fig. Sugimoto A. et al.
B
A
Patients with R0 curative operation (n=98)
All patients (n=110)
Tks5-negative (n=18)
1.0
1.0
Tks5-negative (n=20)
p=0.29
p=0.192
0.8
0.8
Tks5-positive (n=80)
Tks5-positive (n=90)
0.6
0.6
PRFS rate
Overall survival
0.4
0.4
0.2
0.2
0.0
0.0
0
12
24
36
0
12
24
36
Months after surgery
Months after surgery
