# Supplementary material for: The clinicopathologic significance of Tks5 expression of peritoneal mesothelial cells in gastric cancer patients
Source: PLoS One. 2021 Jul 13;16(7):e0253702. doi: 10.1371/journal.pone.0253702 (PMC8277061; doi:10.1371/journal.pone.0253702)
Supplement: S1 Table — Abbreviation: PMC, Peritoneal mesothelial cells. (DOCX) [file pone.0253702.s003.docx]

**S1 Table. Tks5 expression of peritoneal mesothelial cells among 110 gastric cancer cases and 17 healthy cases.**

|  | Tks5 expression of PMCs | |  |
| --- | --- | --- | --- |
| Variables | Positive | Negative | *p* value |
| Gastric cancer cases |  |  |  |
| (N = 110) | 71 (64.5 %) | 39 (35.5 %) | 0.022 |
| Healthy cases |  |  |  |
| (N = 17) | 6 (35.3 %) | 11 (64.7 %) |  |
| Abbreviation: PMC, Peritoneal mesothelial cells | | | |
