# Supplementary material for: The clinicopathologic significance of Tks5 expression of peritoneal mesothelial cells in gastric cancer patients
Source: PLoS One. 2021 Jul 13;16(7):e0253702. doi: 10.1371/journal.pone.0253702 (PMC8277061; doi:10.1371/journal.pone.0253702)
Supplement: S2 Table — * Fisher’s exact test. Abbreviation: IQR, Interquartile range. (DOCX) [file pone.0253702.s004.docx]

**S2 Table. Postoperative recurrence of 98 gastric cancer patients based on Tks5 expression on gastric cancer cells.**

| Variables | Tks5 expression on GC cell | |  |
| --- | --- | --- | --- |
|  | Positive | Negative | *p* |
|  | (n=80) | (n=18) | value |
| All recurrence |  |  |  |
| Present | 14 (17.5 %) | 3 (16.7 %) | 1* |
| Absent | 66 (82.5 %) | 17 (83.3 %) |  |
| Peritoneal recurrence | |  |  |
| Present | 11 (13.8 %) | 1 (5.6 %) | 0.457* |
| Absent | 69 (86.2 %) | 17 (94.4 %) |  |
| * Fisher's exact test |  |  |  |
| Abbreviation: IQR, Interquartile range | | |  |
