# Supplementary material for: The clinicopathologic significance of Tks5 expression of peritoneal mesothelial cells in gastric cancer patients
Source: PLoS One. 2021 Jul 13;16(7):e0253702. doi: 10.1371/journal.pone.0253702 (PMC8277061; doi:10.1371/journal.pone.0253702)
Supplement: Attachment — Submitted filename: PloS One review II (1).doc [file pone.0253702.s006.doc]

**Description**

Peritoneal dissemination is a frequent metastatic pattern of gastric cancer. Peritoneal mesothelial cells activated by gastric cancer cells play an important role in peritoneal carcinomatosis by providing the invasive front. The current work relies on a previously published report on an adaptor protein, Tks5. Tks5 activity has been shown to facilitate peritoneal mesothelial cell migratory capacity and invasiveness. In the current work, the authors have investigated the clinicopathological correlation of Tks5 in PMC in a gastric cancer patient cohort.

1) General comments:

The overall flow of the paper is satisfactory. However, the authors are encouraged to address the concerns and revise their manuscript before resubmission.

2) Detailed comments:

Abstract:

Abstract is compact and easy to understand

Introduction:

Satisfactory and easy to understand

Materials and methods:

satisfactory

Results:

Authors are encouraged to address the following concerns

1. Provide a better image/improved staining quality for Tks5
2. Functional assay to provide insight on how Tks5 is activated in PMC- whether GC cells induce the expression in PMC? What type of stimulus can activate Tks5 expression in PMC?
3. What is the expression status of Tks5 in healthy peritoneum?
4. Functional data to identify Tks5 dependent gene regulation. How the overall gene landscape is altered in absence of Tks5. Does Tks5 regulate EMT gene signatures or other markers that can affect immune cells in the TME?
5. Does Tks5 play any role in chemoresistance or angiogenesis?

Discussion:

Based on current data, it is easy to understand

Conclusion:

Based on the current data provided, it is easy to understand

References:

good

Images/tables (if any):

Not satisfied with the staining quality. Authors are encouraged to provide a better image/improved staining quality in the revised version.

Language quality:

Easy to understand. Good flow throughout the text. There are a few typographical/grammar errors. Authors are encouraged to correct them in the revised version of the manuscript.

Statistic results (if applicable):

Satisfactory
